# Characteristics and outcome of congenital mesoblastic nephroma: A report of 376 patients registered in the SIOP 93-01, SIOP WT 2001, UK-IMPORT, and AIEOP protocols

**DOI:** 10.1371/journal.pone.0349345

**Published:** 2026-05-26

**Authors:** Paola Quarello, Rana Dandis, Tanzina Chowdhury, Arnauld Verschuur, Jesper Brok, Gordan Vujanic, Christian Vokuhl, Paola Collini, Aurore Coulomb, Annalisa Serra, Davide Biasoni, Paula Flores, Leo Kager, Heidi Segers, Karolina Malić Tudor, Marta Maria Burman, Nuno Jorge dos Reis Farinha, Patrik Romerius, Jan Godzinski, Martine van Grotel, Gema Lucia Ramirez-Villar, Beatriz De Camargo, Rhoikos Furtwängler, Reem Al-Saadi, Harm Van Tinteren, Norbert Graf, Filippo Spreafico, Marry van den Heuvel-Eibrink

**Affiliations:** 1 Pediatric Onco-Hematology, Stem Cell Transplantation and Cellular Therapy Division, Regina Margherita Children’s Hospital, Turin, Italy; 2 Department of Pediatric and Public Health Sciences, University of Turin, Turin, Italy; 3 Princess Máxima Centre for Pediatric Oncology, Utrecht, The Netherlands; 4 Department of Hematology and Oncology, Great Ormond Street Hospital for Children NHS Foundation Trust, London, United Kingdom; 5 La Timone, Children’s Hospital, Pediatric Oncology Department, APHM, Marseille, France; 6 Department of Pediatrics and Adolescent Medicine, Rigshospitalet, Copenhagen, Denmark; 7 Department of Pathology, Sidra Medicine, Doha, Qatar; 8 Department of Pathology, Division of Pediatric Pathology, University of Bonn, Germany; 9 Soft Tissue Tumour Pathology Unit, Fondazione IRCCS Istituto Nazionale dei Tumori, Milan, Italy; 10 Sorbonne Université, AP-HP, Hôpital Trousseau, Service d’Anatomie et Cytologie pathologiques, Paris, France; 11 Bambino Gesù Children’s Hospital IRCCS, Onco-hematology Unit, Rome, Italy; 12 Surgical Department, Pediatric Surgery Unit, Fondazione IRCCS Istituto Nazionale dei Tumori, Milan, Italy; 13 Servicio de Cirugía General, Hospital de Pediatría S.A.M.I.C Prof. Dr. Juan P. Garrahan, Buenos Aires, Argentina; 14 Department of Pediatrics and Adolescent Medicine (LK), St. Anna Children’s Hospital, Medical University, Vienna, Austria; 15 Department of Pediatric Hemato-Oncology, Catholic University Leuven, Leuven, Belgium; 16 Department of Pediatric Hematology and Oncology, University Hospital Split, University of Split School of Medicine, Split, Croatia; 17 Department of Pediatric Oncology and Hematology, Division of Pediatric and Adolescent Medicine, Oslo University Hospital, Oslo, Norway; 18 University Hospital Centre São João (CHUSJ) - Pediatric Oncology Service, Porto, Portugal; 19 Department of Pediatrics, Skåne University Hospital, Lund, Sweden; 20 Department of Pediatric Surgery, Marciniak Hospital, Wroclaw, Poland; 21 Department of Pediatric Traumatology and Emergency Medicine, Medical University, Wroclaw, Poland; 22 Virgen del Rocío University Hospital, Department of Pediatric Oncology, Sevilla, Spain; 23 Grupo Brasileiro de Tumores Renais (Brazilian Renal Tumour Group), São Paulo, Brazil; 24 Division of Pediatric Hematology and Oncology, Department of Pediatrics, Inselpital University Hospital, Bern, Switzerland; 25 Department of Pediatric Hematology and Oncology, Saarland University, Homburg, Germany; 26 University College London, Developmental Biology and Cancer Dept, London, United Kingdom; 27 Department of Histopathology, Great Ormond Street Hospital for Children NHS Foundation Trust, London, United Kingdom; 28 Paediatric Oncology Unit, IRCCS Istituto Giannina Gaslini, Via Gaslini 5, Genoa, Italy; 29 Division of Childhealth, Wilhelmina Children’s Hospital, University of Utrecht, The Netherlands; Institute of Public Health from Guanajuato State, MEXICO

## Abstract

**Background:**

Congenital mesoblastic nephroma (CMN) is the most common renal neoplasm diagnosed in the very first months of life. Complete nephrectomy only is the gold standard treatment.

**Objectives and methods:**

This retrospective study aimed to explore the characteristics and outcome of CMN patients registered in the SIOP 93–01, SIOP WT 2001, UK-IMPORT, and AIEOP studies (1993–2019).

**Results:**

A total of 376 CMN cases were identified, with a median age at diagnosis of 28 days. Stage information was available for 337 patients: 92/337 (27.4%) were diagnosed with stage I, 177/337 (52.6%) with stage II, and 67/337 (20%) with stage III. Among 272 patients with available histological data, 113/272 (41.5%) had classic, 105/272 (38.6%) cellular, and 54/272 (19.9%) mixed subtype. Treatment details were available for 314 patients; 248 (79%) underwent initial surgery, and 66 (21%) received preoperative chemotherapy. Among the latter group, 60% of patients showed a measurable reduction in tumor volume, indicating a favorable response to chemotherapy.

The 5-year event-free survival rate was 93.8%, and the overall survival rate was 96.9%. The cumulative 5-year incidence of relapse was 5.3%, with a median time to recurrence of 4 months. Of the 16 relapse cases, 8 were in the cellular subtype, 5 in the mixed subtype, and 3 in the classical subtype.

**Conclusions:**

This study confirms that CMN patients have an excellent outcome, with complete surgical resection being curative in the majority of cases. Chemosensitivity is observed in a significant proportion, suggesting that neoadjuvant chemotherapy may be a viable option in selected cases. While age at diagnosis, histological subtype, and survival outcomes are consistent with previous reports, we highlight that recurrences, though infrequent, tend to occur early and are not restricted to the cellular subtype. Further prospective studies and molecular investigations are required to refine clinical management strategies and update treatment recommendations.

## Introduction

Congenital mesoblastic nephroma (CMN) is the most common renal neoplasm diagnosed in the first month of life comprising approximately 3% of all pediatric renal tumours [1–6]. CMN is often diagnosed prenatally, and it can cause severe obstetric and neonatal complications [3,7]. Except for three reports of CMN in patients with Beckwith-Wiedemann syndrome, no association with genetic predisposition syndromes had been reported so far [1,3,8].

CMN is classified histologically into classic, cellular and mixed subtypes based on a different morphology [9–11]. Recurrent genetic aberrations reported in CMN include chromosomal translocation t(12;15)(p13;q25), resulting in a fusion of the *ETV6* and *NTRK3* genes [6,12,13]. More recent studies have identified other molecular alterations in CMN, including *NTRK1*, *NTRK2*, *BRAF*, *RET*, *MET*, and *NTRK3* with other partners fusions, and *BRAF* internal tandem duplications (ITDs) [13–15]. Classical CMN was shown to harbor *EGFR*-ITDs [14,15]. The molecular profile of mixed CMN is less defined.

The vast majority of CMN patients presented with localized disease at diagnosis (stage I/II), with stage III documented in ~17% of patients, while histologically proven stage IV and stage V patients have never been reported with *de novo* CMN, to our knowledge [3].

Only a few studies reporting on CMN have been based on series from national or international registries [2,4]. The largest reported cohort, comprising 535 children, has been recently published by the Children’s Oncology Group (COG) and includes both patients enrolled in the AREN03B2 protocol (2004–2019) and a historical cohort of CMN patients (1973–2001) [16].

To date, no reports of CMN have been published from the protocols of the International Society of Pediatric Oncology – Renal Tumour Study Group (SIOP-RTSG) or the Italian Association of Pediatric Hematology and Oncology (AIEOP). The aim of the current retrospective study was to describe a cohort of CMN patients enrolled in multinational studies prior to the initiation of the prospective CMN registration in the SIOP-RTSG-2016 UMBRELLA study in 2019. This collaborative study focuses on the description of CMN clinical characteristics, histology, treatment and outcomes.

## Patients and methods

### Patients

All patients diagnosed with histologically proven CMN and registered in the SIOP 93‐01 [1994‐2001], SIOP WT 2001 [2002‐2018], AIEOP [Italian hospital-based registry of pediatric cancer (Mod 1.01) [17,18], AIEOP CNR-92: 1993–2002, AIEOP TW-2003: 2003–2016], and UK‐IMPORT (United Kingdom Improving Population Outcomes for Renal Tumours of childhood) [2012‐2019] databases were included. Some of the included patients had been already reported in previously published national reports [2–4]. Informed consent for registration in the SIOP 93‐01, SIOP WT 2001, UK‐IMPORT, AIEOP protocols including AIEOP Mod 1.01 study was obtained from parents of included pediatric patients, according to national law and regulations. Databases were merged centrally by the SIOP-RTSG office, and variables and codes were translated into a set of consistent anonymized variables.

All listed clinical protocols were primarily focused on Wilms tumour (WT) patients but allowed prospective registration of CMN cases and collection of observational clinical, pathological and outcome data. Molecular data were not systematically collected. Due to the rarity of this tumour, we also included all patients with a confirmed CMN diagnosis registered in the Italian hospital-based registry of pediatric cancer that had been captured at the time of the above mentioned AIEOP protocols. For each patient, basic clinical data were collected, including date of diagnosis, tumour site, histological subtype, stage, treatment, and follow up data. In both SIOP and AIEOP protocols, tumor samples were first evaluated by local pathologists at each participating center following standardized criteria defined by the SIOP classification [19]. In addition, to ensure consistency and uniformity in diagnostic classification, cases underwent national central pathology review by expert reference pathologists with specific expertise in pediatric renal tumors. When data were incomplete, every effort was made to query the national coordinators for missing items and to gather more information.

Preoperative chemotherapy and delayed nephrectomy were recommended for all patients with renal tumours diagnosed over the age of 7 months, as per the SIOP and AIEOP protocols. The indication for primary surgery in children under 7 months of age, based on the higher likelihood of non-WT, was progressively incorporated and more clearly formalized in the 2008 guideline update [5]. Therefore, this age-based distinction was not uniformly applied across all protocols included in the study but reflects a refinement introduced in later guidelines.

### Statistical analysis

The study used summary statistics (medians, ranges: minimum-maximum) for continuous variables and presented categorical data as numbers and percentages. Comparative analyses were conducted with parametric and non-parametric tests to compare variables across histology types and treatment strategies.

Kaplan-Meier methods estimated overall (OS) and event-free survival (EFS) rates post-diagnosis, with comparisons made using the log-rank test. Five-year survival rates were reported with 95% confidence intervals. Cumulative Incidence of Relapse (CIR) was calculated using the competing risks approach, considering non-relapse mortality as a competing event. The analysis was conducted using the Fine and Gray sub distribution hazard model, with group comparisons performed via Gray’s test. CIR curves were visualized using the cumulative incidence function.

Univariable survival analyses examined factors like gender, age, period of diagnosis, tumour stage, and histological subtype.

Differences in tumour volume reduction across histological subtypes after preoperative chemotherapy were evaluated using paired t-tests and a linear mixed-effects model.

Statistical software used included R version 4.4.2 and IBM SPSS Statistics.

## Results

### Clinical features

In total, 376 patients with a CMN were registered between 1993 and 2019. 104/376 (28%) were diagnosed in the period between 1993 and 2001, 154/376 (41%) between 2002 and 2010, and 118/376 (31%) after 2011. These consisted of 221 males, and 155 females (1.4:1 ratio).

**Fig 1:**
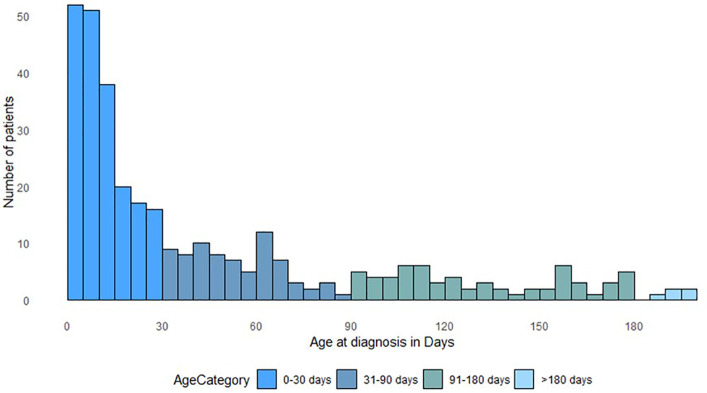
Distribution of CMN patients by age at diagnosis.

The median age at presentation was 28 days (range: 0–953 days). Most patients (194/376, 51.5%) were diagnosed in the first month of life (including 50/376, 13.3%, who were diagnosed at birth), followed by 75 (20%) patients aged between 1 and 3 months, 62 (16.5%) between 4 and 6 months, and 45 (12%) patients diagnosed beyond the age of 6 months (Fig 1). The comparison of clinical details by age at diagnosis (younger or older than 6 months) is reported in **S1 Table**.

One patient harboured trisomy 21; no other germline alterations were documented in the cohort.

Information on stage was available for 337/376 patients (89.6%). Stage I was documented in 92/337 (27.4%) of patients, stage II in 177/337 (52.6%) of patients and stage III disease in 67/337 (20%) of patients. One stage IV patient was diagnosed as CMN. She was a 10-month-old female patient diagnosed in 1994 with liver metastasis at presentation, a local stage III tumour, and an unknown histological subtype. She received neoadjuvant chemotherapy with Actinomycin D and Vincristine (AV), followed by complete nephrectomy and subsequent adjuvant chemotherapy (Epirubicin/Carboplatin/Ifosfamide/Etoposide). At last long-term follow up, she was alive having never experienced a relapse. Stage V patients were not observed.

Information on histological subtype was available for 272/376 patients (72%). It included 113/272 (41.5%) patients with classical, 105/272 (38.6%) with cellular, and 54/272 (19.9%) with mixed subtype. No difference in gender among the histological subtypes was observed. The median age at diagnosis was 13 days (0–953 days) for patients with classical subtype, 60 days (0–850 days) for patients with the cellular subtype, and 30 days (0–393 days) for those with the mixed subtype (*p < 0.001*).

The median tumour volume at diagnosis and the median tumour weight at surgery (in patients who underwent upfront surgery) was 43 ml and 54 g for classical, 297 ml and 201 g for cellular, and 149 ml and 118 g for mixed subtype, respectively (*p < 0.001*). No association between histological type and tumour stage was observed (Table 1).

**Table 1:** Clinical characteristics by histological subtypes.

Characteristic	Overall N = 376^*1*^	Cellular N = 105^*1*^	Classical N = 113^*1*^	Mixed N = 54^*1*^	Unknown N = 104^*1*^	p-value^*2*^
Age at diagnosis *(days)*	28 (0-953)	60 (0-850)	13 (0-953)	30 (0-393)	28 (0-757)	*<0.001*
						
Gender						*0.6*
Female	155 (41%)	44 (42%)	43 (38%)	18 (33%)	50 (48%)	
Male	221 (59%)	61 (58%)	70 (62%)	36 (67%)	54 (52%)	
						
Tumour Weight at surgery* *(g)*	91 (8-895)	201 (10-895)	54 (8-171)	118 (38-780)	212 (23-460)	*<0.001*
Unknown	82	22	31	12	17	
						
Tumour Volume at Diagnosis *(ml)*	92 (3 –1,412)	297 (6 –1,061)	43 (3-868)	149 (9 –1,412)	67 (4-690)	*<0.001*
Unknown	138	27	24	15	72	
						
Stage						*0.8*
Stage I	92 (27.4%)	34 (33.4%)	24 (21.2%)	4 (7.4%)	30 (44%)	
Stage II	177 (52.6%)	48 (47%)	67 (59.3%)	37 (68.5%)	25 (37%)	
Stage III	67 (20%)	20 (19.6%)	22 (19.5%)	13 (24.1%)	12 (17.5%)	
Unknown	40	3	0	0	37	

^*1*^Median (Min, Max); n (%), ^*2*^ Kruskal-Wallis rank sum test; Pearson’s Chi-squared test**.** Unknowns are excluded from tests**.** *including only patients who underwent upfront surgery.

### Treatment

Treatment details were available for 314 patients: 248/314 (79%) patients underwent initial surgery, while 66/314 (21%) received preoperative chemotherapy (Table 2). Complete nephrectomy was performed in all patients; no partial nephrectomy was documented.

**Table 2:** Characteristics between CMN patients treated with preoperative chemotherapy compared to those with frontline surgery.

Characteristic	N	Overall N = 376^*1*^	Direct surgery N = 248^*1*^	Preoperative chemotherapy N = 66^1^	Unknown N = 62^*1*^	p-value^*2*^
Age *(days)*	376	28 (0-953)	23 (0-839)	94 (0-953)	28 (1-850)	<0.001
Gender	376					0.2
Female		155 (41%)	91 (37%)	31 (47%)	33 (53%)	
Male		221 (59%)	157 (63%)	35 (53%)	29 (47%)	
Period of diagnosis	376					0.4
1993_2001		104 (28%)	47 (19%)	12 (18%)	45 (73%)	
2002_2010		154 (41%)	109 (44%)	35 (53%)	10 (16%)	
2011_2019		118 (31%)	92 (37%)	19 (29%)	7 (11%)	
Stage	376					0.3
Stage I		92 (27.4%)	59 (24.5%)	20 (32%)	13 (39.4%)	
Stage II		177 (52.6%)	135 (56%)	26 (42%)	16 (48.5%)	
Stage III		67 (20%)	47 (19.5%)	16 (26%)	4 (12.1%)	
Unknown		40	7	4	29	
Tumour Weight at surgery *(g)*	238	106 (8 –1,602)	91 (8-895)	190 (15 –1,602)	100 (36-540)	<0.001
Unknown		139	83	19	37	
Volume at Diagnosis *(ml)*	240	85 (3 –1,412)	80 (3 –1,412)	232 (8-888)	97 (32-175)	0.007
Unknown		138	71	11	56	
Histological subtype	376					0.8
Cellular		105 (38.6%)	84 (37.7%)	19 (42.2%)	2 (50%)	
Classical		113 (41.5%)	96 (43%)	17 (37.8%)	0 (0%)	
Mixed		54 (19.9%)	43 (19.3%)	9 (20%)	2 (50%)	
Unknown		104	25	21	58	

^*1*^Median (Min, Max); n (%), ^*2*^Kruskal-Wallis rank sum test; Pearson’s Chi-squared test. Unknowns are excluded from tests.

The patients who received preoperative chemotherapy were significantly older at diagnosis (94 days versus 23 days, *p < 0.001*), higher tumour volume at diagnosis and larger tumour weight at surgery in comparison to patients who underwent upfront surgery (232 ml versus 80 ml, *p = 0.007*, and 190 g versus 91 g, *p < 0.001*). No differences regarding gender, period of diagnosis, histological subtype, and staging were found (Table 2).

**Fig 2:**
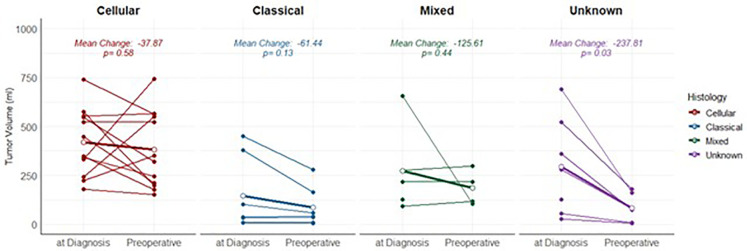
Response to preoperative chemotherapy in selected cases.

Information on volume response after the administration of preoperative chemotherapy was available for 30/66 patients. 60% (18/30) of these patients showed a tumour volume reduction compared to volume at diagnosis, whereas 13.3% (4/30) and 26.7% (8/30) showed a stable or increased tumour volume, respectively (Table 3). No significant association between histological subtype and response to preoperative chemotherapy in terms of tumour volume reduction was observed (*p = 0.22*) (Table 3, Fig 2).

**Table 3:** Tumour response in selected CMN patients that received preoperative chemotherapy (n = 66).

Gender	Age at diagnosis *(months)*	Stage	Histological subtype	Tumour volume at diagnosis *(ml)*	Preoperative volume *(ml)*	Volume response *(ml)*	Volume variation *(%)*
**Patients with chemotherapy response (stability/reduction of tumour volume)**							
Male	3	Stage III	Mixed	657	106	−551	−84
Male	8	Stage I/II	Unknown	690	161	−530	−77
Male	3	Stage I/II	Cellular	574	199	−375	−65
Female	1	Unknown	Unknown	523	179	−344	−66
Female	6	Stage I/II	Unknown	360	75	−285	−79
Male	24	Stage III	Cellular	447	211	−236	−53
Male	13	Stage I/II	Cellular	547	319	−228	−42
Female	32	Stage I/II	Classical	378	163	−215	−57
Male	6	Unknown	Unknown	278	79	−200	−72
Male	15	Stage I/II	Cellular	740	563	−176	−24
Male	15	Stage I/II	Classical	452	280	−172	−38
Female	12	Stage I/II	Cellular	347	175	−172	−50
Male	15	Stage I/II	Cellular	341	245	−96	−28
Male	1	Stage I/II	Unknown	56	8	−48	−85
Male	8	Stage I/II	Classical	103	59	−44	−43
Male	6	Stage I/II	Cellular	179	151	−29	−16
Male	3	Stage I/II	Unknown	26	5	−21	−80
Male	8	Stage I/II	Classical	10	6	−4	−39
Female	0	Stage III	Classical	38	37	−1	−2
Male	4	Stage I/II	Classical	10	10	0	0
Female	9	Stage I/II	Mixed	217	217	0	0
Female	1	Stage I/II	Cellular	523	523	0	0
**Patients without chemotherapy response (increase of tumour volume)**							
Female	0	Stage III	Classical	35	40	+5	+15
Male	2	Stage I/II	Cellular	554	565	+10	+2
Female	13	Stage I/II	Mixed	276	298	+22	+8
Male	1	Stage III	Mixed	92	118	+26	+29
Male	0	Stage I/II	Cellular	223	352	+129	+58
Female	24	Stage III	Cellular	242	551	+309	+128
Female	0	Stage I/II	Cellular	333	742	+409	+123
Male	3	Stage III	Mixed	888	1378	+490	+55

Regarding postoperative treatment, we highlighted the stage III subgroup (n = 67) separately. 9/52 (17.3%) patients with available information had received adjuvant chemotherapy (6 cellular, 2 mixed, 1 unknown subtype) (S2and S3 Tables).

### Outcome

**Fig 3:**
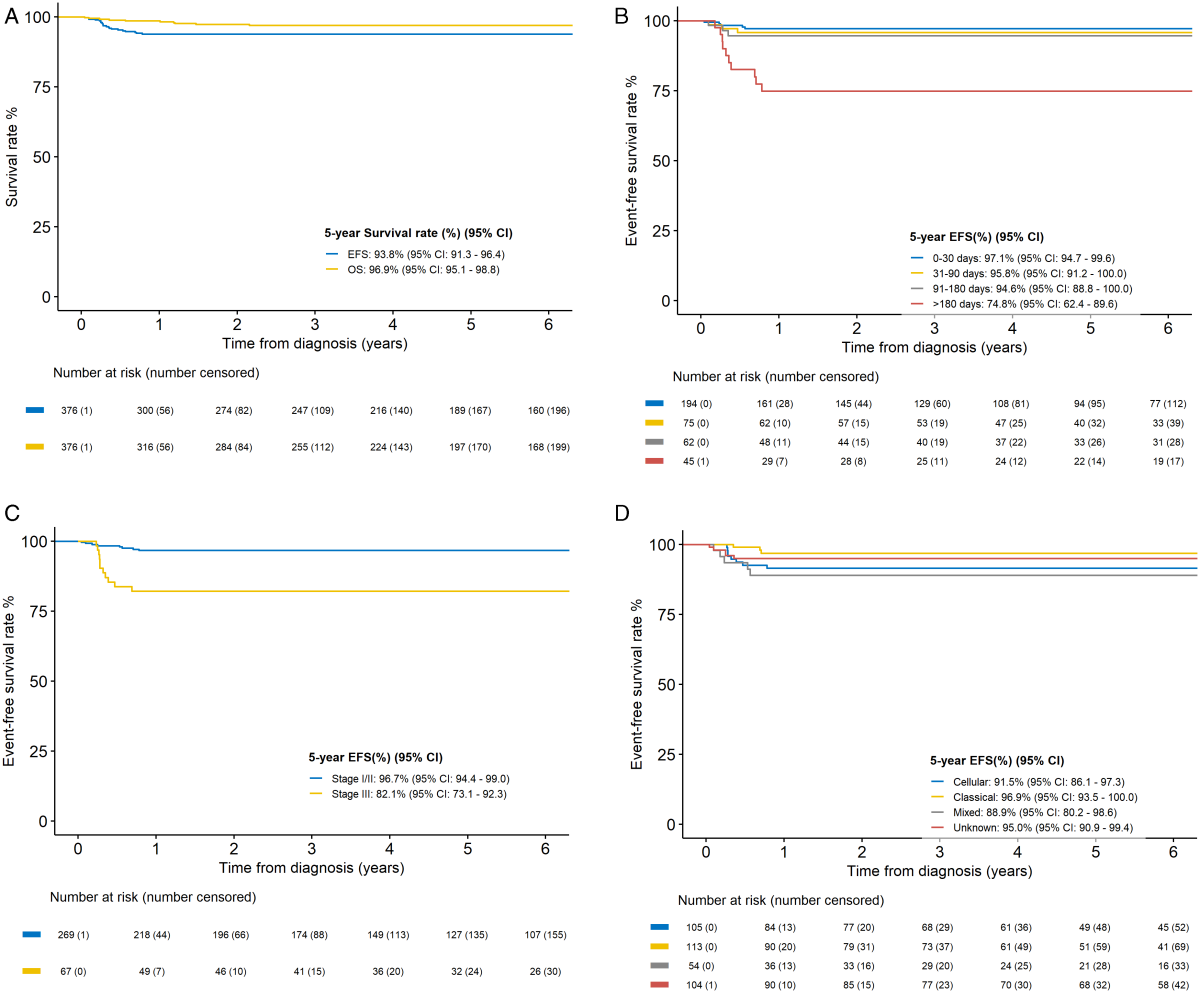
Outcome of CMN patients. 3A. Overall EFS and OS. 3B. EFS by age at diagnosis. 3C. EFS by stage. 3D. EFS by histological subtype.

The median follow up was 5.3 years (range: 0.02-25.8 years). The 5-year EFS rate was 93.8% (95% CI, 91.3-96.4), with a 5-year OS rate of 96.9% (95% CI, 95.1-98.8) (Fig 3A).

Univariate analyses showed that patients older than 6 months at diagnosis had a significantly inferior EFS and OS as compared to those diagnosed before 1 month, between 1 and 3 months or between 3 and 6 months at diagnosis (EFS: 74.8% vs 97.1% vs 95.8% vs 94.6%, *p < 0.0001*; OS: 84.6% vs 98.8% vs 98.6% vs 98.2%, *p < 0.001*). (S4 and S5 Tables, Fig 3B).

In univariate analysis, the EFS and OS for stage I, stage II and vs III were 97.6% vs 96.2 vs 82.1% *(p < 0.0001)* and 98.7% vs 98.1% vs 93.2% *(p = 0.031)*, respectively (S4 and S5 Tables, Fig 3C).

Gender, histological subtype and period of diagnosis did not have a significant association with EFS and OS (S4 and S5 Tables, Fig 3D).

The 5-year CIR was 5.3% (95% CI, 3.7-8.8). Overall, 18 patients experienced a relapse (S6 Table). The site of relapse was unknown in two patients (2/18). Among the remaining 16 patients with available data (16/18), most (12/16) had local relapse, while two had pulmonary relapse, one had liver recurrence, and one had testicular recurrence.

The median time to recurrence was 3.6 months (range: 1-9.5 months). Two relapses occurred among the 92 patients with stage I (2.2%), five among the 177 patients with stage II (2.8%), and eleven among the 67 patients with stage III (16.4%). 8/16 relapses with available data occurred in cellular subtype, 5/16 in mixed and 3/16 in classical subtypes (S6 Table). In the overall cohort, relapses occurred in 8/105 patients with the cellular subtype (7.6%), in 5/54 with the mixed subtype (9.3%), and in 3/113 with the classical subtype (2.7%) (S1 Fig). Treatment administered at relapse is reported in S6 Table.

At the time of the last follow-up, 11 patients had died. Disease progression after relapse was the cause in six patients. Deaths due to treatment-related events were reported in two patients (sepsis after preoperative chemotherapy and post-surgical complication). One patient experienced a cardiac failure following a subsequent diagnosis of adrenocortical carcinoma and another patient died from Sudden Infant Death Syndrome after experiencing a previous relapse. One patient’s cause of death was unknown.

## Discussion

CMN is a very rare tumour, which represents approximately 3% of all pediatric renal tumours (i.e., 10–20 cases annually across Europe). There are rare studies on CMN with a substantial numbers of patients based on international/national registries [2–4,16].

No differences in sex ratio among the histological subtypes were seen in our cohort. Previous studies reported a slight predominance of males among CMN consistent with our observation [20].

Here, we reported for the first time a 1-month -old female patient with mixed type, stage III CMN and Down syndrome. Previously, Beckwith-Wiedemann syndrome was reported in three CMN patients as the only genetic predisposition syndrome that appeared to be associated with the condition [1,3,8]. In addition, one single CMN patient was reported with isolated hemihypertrophy, and two others presented with complex urogenital malformations [4,21]. As it was not the intention of the registries to document germline alterations in CMN, and phenotypic data were not captured completely, it is conceivable that phenotype-genotype correlations are underestimated, and certainly prospective cohort studies will need to provide more accurate data.

The median age at diagnosis in our cohort was 28 days, consistent with findings reported in the literature [2,4,16]. In 71.5% of the patients CMN was diagnosed in the first 3 months of life and in 88% of cases by 6 months of life. The young age of patients with CMN supports the fact that although WT is the most commonly found renal tumour overall diagnosed over the age of 3 months, the prevalence of CMN in the first two months of life exceeds that of WT previously reported in a study of 750 infants with renal tumours [5].

The oldest patient in our cohort was diagnosed at 953 days (31.3 months) of age in 1994, which is similar to the median age of patients in the AREN03B2 (25 months) and GPOH (31.3 months) cohorts. In the historical NWTS cohort, the oldest patient was 48 months of age, but one has to take into consideration the fact that this cohort was from the time period (1973–2001) when other tumours, such as metanephric stromal tumours, histologically very similar to CMN, were not known [22].

Because of its tendency for infiltrative growth into the renal sinus or perirenal fat, stage I CMN occurred significantly less frequently than stage II in our cohort and in other studies [2,4]. Most patients in our study had stage II tumour (52.6%), which is similar to other bigger series including 48% in GPOH series [4], 51% in AREN03B2 series [16], and 47% in the historical NWTS series [16], but significantly fewer than in the UK series (70%) [2]. In contrast, in all studies stage III was diagnosed in ~20% of patients [2,4,16].

In our cohort, we describe one patient with stage IV disease and liver metastases that were not histologically proven; furthermore, even though a central pathology review was performed, the diagnosis was made in 1994, before other renal entities were recognised, and since histological slides were not available for a re-review, this case should be taken with some doubt.

There were no patients with stage IV CMN in the GPOH or UK series [2,4], but rare alleged stage IV cases have been reported including one patient in AREN03B2, and two patients in the historical NWTS series, as well as one patient in the comprehensive review of the literature [3,16]. The latter case was not histologically proven stage IV, and the case from the AREN03B2 cohort was subsequently dismissed as stage IV (alleged lung metastases were on a radiological re-review interpreted as atelectasis). Finally, two alleged stage IV cases in the historical NWTS cohort were diagnosed before other renal entities were recognised [22], and no clinical or pathological details about these cases were provided, so they should be regarded as inconclusive at best [16].

In the current study, the cellular (105/272, 38.6%) and classic (113/272, 41.5%) subtypes occurred at similar prevalence, which was comparable to the UK study, but significantly differed from the AREN03B2 cohort (cellular subtype 83/136, 61%; classic subtype 30/136, 22%) [2,16]. The mixed type prevalence was similar in all three studies (~20%) [2,16].

In our cohort, patients with cellular CMN were significantly older at diagnosis, had a significantly higher tumour volume at diagnosis, and a tumour weight at surgery than patients with classical CMN, as previously reported [3,4,16]. These clinical differences are consistent with the hypothesis that cellular CMN may represent a biologically more aggressive subtype. Unfortunately, molecular data that could further explore this interpretation—by providing biological correlates of these clinical features—are not available in the described cohort.

Radical nephrectomy and the removal of the perirenal fat is the gold standard of treatment for CMN, and the surgical margins should be wide due to the tendency of CMN to infiltrate the surrounding perirenal fat. Lymph node sampling is also recommended as for all other renal tumours [2,4,16,23].

It is generally agreed that upfront nephrectomy is recommended for renal tumours in patients under 7 months of age due the high chance of non-WT tumours [3,5,24]. Preoperative chemotherapy may however be warranted in a subset of these young patients when imaging or clinical assessment suggests a high risk of complications during surgery and anaesthesia.

Interestingly, in a subgroup of 30 patients for whom we had response to neoadjuvant chemotherapy data, in 60% a significant reduction in tumour size was observed, with no association to histological subtype. In the remaining 40%, either stability or an increase in tumour size was noted. We cannot exclude the possibility that CMN exhibits even higher chemosensitivity than observed, as we lacked certain histological information, such as the percentage of necrotic tissue, which could help explain the observed increase in tumour size. A significant increase in tumour size was reported in one CMN patient who experienced a doubling of tumour volume after preoperative chemotherapy but this was found to be predominantly necrotic (80%) upon pathological examination [4].

Our data demonstrated chemosensitivity in a high percentage of patients, which can be helpful in the decision-making process, as it is crucial to carefully balance the risks of surgery with preoperative chemotherapy. This is particularly important considering that, in the past, the main causes of death in very young infants were surgery-related [3], as it was the case in two patients in our cohort. It needs to be mentioned that these two patients deceased more than 25 years ago, when both diagnostic techniques and supportive treatment were certainly less available and less advanced. Carefully balancing the related risks of given treatment (either surgery or chemotherapy) is important, and treatment in specialized centres with renal tumour surgery expertise is therefore advised, even though CMN is considered a tumour with a low malignant potential.

In current clinical practice, patients with CMN stage III pose a significant challenge with regards to therapy choices in the context of adjuvant chemotherapy. The literature review revealed that surgery alone seems to be sufficient for these patients, especially for classical or mixed histological subtypes. The use of additional chemotherapy might reduce the risk of relapse in patients with stage III cellular CMN, but the data from our study and the literature so far do not allow us to support this possible benefit [3]. Also, even when they relapse, most patients can be salvaged with therapy administered at recurrence. In our cohort, 9/52 (17.3%) patients with stage III received adjuvant chemotherapy, and 2/9 (22.2%) relapsed, which was not significantly different from the patients who received no post-operative chemotherapy (14/43, 32.5% relapsed). Similarly, in the AREN03B2 cohort, 13% of the 92 stage II/III cellular/mixed CMNs received adjuvant chemotherapy, with an 8% relapse rate. Among the 80 patients who did not receive adjuvant chemotherapy, 11% relapsed. This suggests that the benefit of adjuvant chemotherapy remains unclear, even for the cellular subtype of CMN [16]. Clinical surveillance and future molecular biomarkers may further guide this decision-making forthcoming.

Overall, we confirm an excellent 5-year EFS (93.8%, 95% CI, 91.3-96.4) and 5-year OS rates (96.9%, 95% CI, 95.1-98.8), consistent with the data from the literature [2–4,16], indicating that most patients can be successfully salvaged upon relapse. Consequently, administering adjuvant therapy to all stage III patients—or even restricting it only to those with cellular subtypes—is not rational.

In our cohort, the 5-year CIR was 5.3% (95% CI, 3.7-8.8), with a median time to recurrence of 4 months. As in other series, relapses were mostly locally and occurred not only in cellular subtype but also in mixed and, less frequently in classical subtype. Based on these observations and considering the radiation risk for these patients, we recommend oncological surveillance for at least 18–24 months after diagnosis, as well as long-term follow-up for late effects associated with nephrectomy and potential chemotherapy.

Literature data reported a relapse in around 4–8% of CMN patients, almost all of which occurred within 12 months after diagnosis [3,4,16] as it was in our cohort, where the latest relapse was 9.5 months after the diagnosis. In the AREN03B2 cohort, 2/10 patients relapsed after 14.2 and 17.9 months after diagnosis, respectively, [16] whereas no other series reported relapses after 12 months. Relapses seem to occur locally with a significant mortality rate in previous series [3,4,16,24–26]. It has been suggested that the risk of recurrence is associated with cellular and mixed subtypes, while for classical CMN, the risk is either absent or, in any case, less than 1% [3,16].

Although older age and stage III appear to have a negative prognostic association, and there may be a trend towards a higher risk of relapse in patients with cellular and mixed subtypes, the small number of events prevented further statistical analysis of the independence of these factors.

Identifying specific recurrent molecular alterations such as NTRK-ETV6 fusion and other tyrosine-kinases receptors rearrangements in CMN may be helpful for targeted therapeutic strategies providing new therapeutic possibilities for the very rare patients with relapsed or metastatic CMN [13,25–28], or for those patients with a large tumour volume and difficult to operate (neoadjuvant setting). If the predictive role of such aberrations had a therapeutic value also in pediatric tumours, they could be valuable potential stratifiers for targeted drugs over conventional low intensity chemotherapy [13–15].

The main strength of this retrospective study is that it is the first cooperative report from the large studies of SIOP-RTSG, UK-IMPORT and AIEOP and one of the largest cohorts of CMN ever reported with an adequate follow up. Our study also has some weaknesses, since the SIOP 93−01, SIOP WT 2001, and AIEOP databases were designed to enrol mainly WT patients, CMN cases may be under-reported. In addition, this retrospective study, which was based on a long period of registration, was hampered by missing data, thereby providing less robust conclusions. Also, SIOP 93−01 cases were not re-reviewed after some new entities similar to CMN were recognised.

The current SIOP-RTSG UMBRELLA 2016 protocol addresses completeness of registration of all renal tumours, central review of radiology and histology, and ongoing molecular characterisation which will conceivably provide data for consensus on clinical management and updated treatment recommendations [29].

In conclusion, our study showed that patients with CMN had an excellent outcome, and that complete surgical removal was curative in most cases. Tumour weight and volume at diagnosis as well as age correlated with histology (cellular CMNs were older and showed larger dimensions). We found that chemosensitivity was present in a significant percentage of cases, suggesting that, in specific inoperable young cases with a high rupture risk, neoadjuvant therapy could be a reasonable option, as 60% of the patients’ response to AV. Additionally, we observed that recurrences occurred early and were not limited to the cellular histological subtype, although they were less frequent in classical CMN, and our findings do not suggest a clear benefit of adjuvant chemotherapy. Further prospective and molecular studies will be needed to provide data for consensus on clinical management and updated treatment recommendations.

## Supporting information

S1 Table Clinical features according to age at diagnosis. (DOCX)

S2 Table Demographic and clinical characteristics of Stage III patients. (DOCX)

S3 Table Characteristics, treatment and outcome of stage III CMN patients. (DOCX)

S4 Table Univariate analysis of determinants associated with 5-Year event-free survival rates. (DOCX)

S5 Table Univariate analysis of determinants associated with 5-Year overall survival rates. (DOCX)

S6 Table Characteristics and survival of CMN patients with recurrent disease. (DOCX)

S1 Fig Cumulative incidence of relapse by age at diagnosis, stage and histological subtype. (PNG)
