# Supplementary material for: Characteristics and outcome of congenital mesoblastic nephroma: A report of 376 patients registered in the SIOP 93-01, SIOP WT 2001, UK-IMPORT, and AIEOP protocols
Source: PLoS One. 2026 May 26;21(5):e0349345. doi: 10.1371/journal.pone.0349345 (PMC13210389; doi:10.1371/journal.pone.0349345)
Supplement: S1 Table — (DOCX) [file pone.0349345.s001.docx]

**Supplementary Table 1. Clinical Features According to Age at Diagnosis**

| Characteristics | N | Overall N = 376*^1^* | Less than 6 months N = 331*^1^* | 6 months or more N = 45*^1^* | p-value*^2^* |
| --- | --- | --- | --- | --- | --- |
| Gender | 376 |  |  |  | 0.7 |
| Female |  | 155 (41%) | 138 (42%) | 17 (39%) |  |
| Male |  | 221 (59%) | 193 (58%) | 29 (63%) |  |
| Tumour Weight at surgery *(g)* | 239 | 112 (10-1,602) | 98 (8-895) | 404 (25-1,602) | <0.001 |
| Unknown |  | 138 | 122 | 18 |  |
| Tumour Volume at Diagnosis *(ml)* | 239 | 92 (3-1,412) | 78 (3-1,048) | 377 (5-1,412) | <0.001 |
| Unknown |  | 138 | 123 | 15 |  |
| Stage | 376 |  |  |  | 0.024 |
| Stage I |  | 92 (27.4%) | 77 (25.8%) | 15 (40.6%) |  |
| Stage II |  | 177 (52.6%) | 168 (56.2%) | 9 (24.3%) |  |
| Stage III |  | 67 (20%) | 54 (18%) | 13 (35.1%) |  |
| Unknown |  | 40 | 32 | 8 |  |
| Histology | 376 |  |  |  | 0.01 |
| Cellular |  | 105 (38.6%) | 85 (35.4%) | 20 (62.5%) |  |
| Classical |  | 113 (41.5%) | 104 (43.4%) | 9 (28.1%) |  |
| Mixed |  | 54 (19.9%) | 51 (21.2%) | 3 (9.4%) |  |
| Unknown |  | 104 | 91 | 13 |  |
| *^1^* Median (Min, Max); n (%) | | | | | |
| *^2^* Wilcoxon rank sum test; Pearson’s Chi-squared test(Unknowns and Stage IV were not used in the tests) | | | | | |
