# Supplementary material for: Characteristics and outcome of congenital mesoblastic nephroma: A report of 376 patients registered in the SIOP 93-01, SIOP WT 2001, UK-IMPORT, and AIEOP protocols
Source: PLoS One. 2026 May 26;21(5):e0349345. doi: 10.1371/journal.pone.0349345 (PMC13210389; doi:10.1371/journal.pone.0349345)
Supplement: S2 Table — (DOCX) [file pone.0349345.s002.docx]

**Supplementary Table 2. Demographic and Clinical Characteristics of Stage III Patients**

| Characteristic | N = 67*^1^* |
| --- | --- |
| Age at diagnosis *(days)* | 41 (0-757) |
| Gender |  |
| Female | 32 (48%) |
| Male | 35 (52%) |
| Histological subtype |  |
| Classical | 22 (40%) |
| Cellular | 20 (36.4%) |
| Mixed | 13 (23.6%) |
| Unknown | 12 |
| Initial treatment |  |
| Direct surgery | 47 (74.6%) |
| Preoperative chemotherapy | 16 (25.4%) |
| Unknown | 4 |
| Postoperative treatment |  |
| no | 43 (82.7%) |
| yes | 9 (17.3%) |
| unknown | 15 |
| Occurrence of Relapse | 11 (16.4%) |
| Final disease status |  |
| Alive | 62 (92.5%) |
| Death | 5 (7.5%) |
| *^1^*Median (Min, Max); n (%)  ^2^95% Confidence interval | |
