# Supplementary material for: Characteristics and outcome of congenital mesoblastic nephroma: A report of 376 patients registered in the SIOP 93-01, SIOP WT 2001, UK-IMPORT, and AIEOP protocols
Source: PLoS One. 2026 May 26;21(5):e0349345. doi: 10.1371/journal.pone.0349345 (PMC13210389; doi:10.1371/journal.pone.0349345)
Supplement: S3 Table — (DOCX) [file pone.0349345.s003.docx]

**Supplementary Table 3. Characteristics, treatment and outcome of stage III CMN patients**

| **Patient** | **Sex** | **Age at diagnosis** *(months)* | **Histological subtype** | **Initial treatment** | **Post-operative treatment** | **Relapse** | **Status at last follow up** | **Follow up**  *(months)* |
| --- | --- | --- | --- | --- | --- | --- | --- | --- |
| 1 | Male | 3 | Cellular | CN | no | no | Alive | 70 |
| 2 | Male | 8 | Cellular | CN | no | no | Alive | 0 |
| 3 | Female | 14 | Cellular | Preop CT | yes | yes (4 months) | DOD | 7 |
| 4 | Male | 4 | Cellular | CN | no | no | Alive | 268 |
| 5 | Female | 6 | Cellular | Preop CT | unknown | yes (3 months) | Alive | 87 |
| 6 | Female | 24 | Cellular | Preop CT | yes | no | TD* | 171 |
| 7 | Male | 0 | Cellular | CN | no | no | Alive | 82 |
| 8 | Male | 0 | Cellular | CN | no | no | Alive | 35 |
| 9 | Male | 24 | Cellular | Preop CT | yes | no | Alive | 2 |
| 10 | Female | 3 | Cellular | CN | yes | no | Alive | 86 |
| 11 | Male | 6 | Cellular | CN | no | yes (3 months) | DOD | 12 |
| 12 | Male | 3 | Cellular | CN | no | yes (6 months) | Alive | 123 |
| 13 | Female | 7 | Cellular | CN | unknown | no | Alive | 47 |
| 14 | Female | 0 | Cellular | CN | yes | no | Alive | 141 |
| 15 | Male | 0 | Cellular | CN | no | no | Alive | 193 |
| 16 | Female | 9 | Cellular | CN | yes | no | Alive | 123 |
| 17 | Female | 0 | Cellular | CN | no | no | Alive | 37 |
| 18 | Female | 2 | Cellular | CN | unknown | yes (3 months) | Alive | 91 |
| 19 | Female | 24 | Cellular | Preop CT | no | yes (3 months) | DOD | 14 |
| 20 | Male | 7 | Cellular | CN | no | yes (24 months) | DOD | 26 |
| 21 | Male | 3 | Classical | CN | no | no | Alive | 76 |
| 22 | Male | 10 | Classical | CN | no | yes (8 months) | Alive | 135 |
| 23 | Female | 0 | Classical | CN | no | no | Alive | 66 |
| 24 | Male | 2 | Classical | CN | no | no | Alive | 89 |
| 25 | Female | 0 | Classical | Preop CT | no | no | Alive | 122 |
| 26 | Male | 0 | Classical | CN | unknown | no | Alive | 0 |
| 27 | Female | 0 | Classical | CN | no | no | Alive | 65 |
| 28 | Male | 4 | Classical | CN | no | no | Alive | 112 |
| 29 | Female | 2 | Classical | CN | no | no | Alive | 110 |
| 30 | Female | 1 | Classical | CN | no | no | Alive | 121 |
| 31 | Female | 0 | Classical | Preop CT | no | no | Alive | 22 |
| 32 | Male | 5 | Classical | CN | no | yes (4 months) | Alive | 23 |
| 33 | Male | 2 | Classical | CN | no | no | Alive | 21 |
| 34 | Female | 4 | Classical | CN | no | no | Alive | 20 |
| 35 | Female | 2 | Classical | CN | no | no | Alive | 31 |
| 36 | Female | 0 | Classical | CN | no | no | Alive | 3 |
| 37 | Female | 0 | Classical | CN | no | no | Alive | 120 |
| 38 | Male | 1 | Classical | CN | no | no | Alive | 43 |
| 39 | Female | 0 | Classical | CN | no | no | Alive | 60 |
| 40 | Male | 0 | Classical | CN | no | no | Alive | 124 |
| 41 | Female | 0 | Classical | CN | no | no | Alive | 61 |
| 42 | Female | 7 | Classical | CN | no | no | Alive | 34 |
| 43 | Male | 1 | Mixed | Preop CT | no | no | Alive | 152 |
| 44 | Male | 3 | Mixed | Preop CT | yes | no | Alive | 126 |
| 45 | Male | 0 | Mixed | CN | yes | yes (5 months) | Alive | 225 |
| 46 | Male | 3 | Mixed | Preop CT | unknown | no | Alive | 71 |
| 47 | Male | 0 | Mixed | CN | no | no | Alive | 60 |
| 48 | Female | 1 | Mixed | CN | no | no | Alive | 0 |
| 49 | Male | 0 | Mixed | CN | no | no | Alive | 11 |
| 50 | Female | 1 | Mixed | CN | no | no | Alive | 1 |
| 51 | Male | 0 | Mixed | CN | no | no | Alive | 94 |
| 52 | Female | 0 | Mixed | CN | no | no | Alive | 48 |
| 53 | Male | 1 | Mixed | Preop CT | unknown | no | Alive | 69 |
| 54 | Female | 3 | Mixed | Preop CT | no | no | Alive | 25 |
| 55 | Female | 1 | Mixed | CN | no | no | Alive | 40 |
| 56 | Female | 1 | Unknown | CN | no | no | Alive | 71 |
| 57 | Female | 0 | Unknown | Preop CT | unknown | no | Alive | 56 |
| 58 | Female | 0 | Unknown | Preop CT | unknown | no | Alive | 248 |
| 59 | Male | 2 | Unknown | CN | unknown | no | Alive | 28 |
| 60 | Male | 1 | Unknown | unknown | unknown | no | Alive | 101 |
| 61 | Male | 25 | Unknown | Preop CT | unknown | no | Alive | 86 |
| 62 | Female | 7 | Unknown | Preop CT | yes | no | Alive | 99 |
| 63 | Male | 1 | Unknown | CN | unknown | yes (3 months) | Alive | 35 |
| 64 | Male | 5 | Unknown | unknown | unknown | no | Alive | 97 |
| 65 | Male | 0 | Unknown | unknown | unknown | no | Alive | 118 |
| 66 | Male | 1 | Unknown | unknown | unknown | no | Alive | 109 |
| 67 | Male | 3 | Unknown | CN | no | no | Alive | 136 |

Legend: CN=complete nephrectomy, Preop CT=preoperative chemotherapy, DOD=death of disease, TD=toxic death, *cardiac failure after a subsequent adenocarcinoma
