# Supplementary material for: Characteristics and outcome of congenital mesoblastic nephroma: A report of 376 patients registered in the SIOP 93-01, SIOP WT 2001, UK-IMPORT, and AIEOP protocols
Source: PLoS One. 2026 May 26;21(5):e0349345. doi: 10.1371/journal.pone.0349345 (PMC13210389; doi:10.1371/journal.pone.0349345)
Supplement: S4 Table — (DOCX) [file pone.0349345.s004.docx]

**Supplementary Table 4. Univariate analysis of determinants associated with 5-Year Event-Free Survival Rates**

| Variables | 5-Year Survival Probability^1^ | Confidence Interval | | Log rank test |
| --- | --- | --- | --- | --- |
|  |  | 95% CI Lower Bound**^1^** | 95% CI Upper Bound**^1^** |  |
| Age at diagnosis |  |  |  |  |
| < 30 days | 97.1 | 94.7 | 99.6 | **<0.0001** |
| 31-90 days | 95.8 | 91.2 | 100.0 |  |
| 91-180 days | 94.6 | 88.8 | 100.0 |  |
| > 180 days | 74.8 | 62.4 | 89.4 |  |
| Gender |  |  |  |  |
| Female | 93.1 | 89.0 | 97.3 | 0.44 |
| Male | 93.7 | 90.4 | 97.2 |  |
| Histological subtype |  |  |  |  |
| Cellular | 91.5 | 86.1 | 97.3 | 0.076 |
| Classical | 96.9 | 93.5 | 100.0 |  |
| Mixed | 88.9 | 80.2 | 98.6 |  |
| Period of diagnosis |  |  |  |  |
| 1993_2001 | 91.3 | 86.0 | 96.9 | 0.31 |
| 2002_2010 | 96.6 | 93.7 | 99.6 |  |
| 2011_2019 | 90.5 | 84.3 | 97.1 |  |
| Stage |  |  |  |  |
| Stage I | 97.6 | 94.3 | 100 | **<0.0001** |
| Stage II | 96.2 | 93.2 | 99.2 |  |
| Stage III | 82.1 | 73.1 | 92.3 |  |
| *^1^* Event-Free Survival probabilities at 5 years with 95% confidence intervals. | | | |  |
