# Supplementary material for: Characteristics and outcome of congenital mesoblastic nephroma: A report of 376 patients registered in the SIOP 93-01, SIOP WT 2001, UK-IMPORT, and AIEOP protocols
Source: PLoS One. 2026 May 26;21(5):e0349345. doi: 10.1371/journal.pone.0349345 (PMC13210389; doi:10.1371/journal.pone.0349345)
Supplement: S5 Table — (DOCX) [file pone.0349345.s005.docx]

**Supplementary Table 5. Univariate analysis of determinants associated with 5-Year Overall Survival Rates**

| Variables | 5-Year Survival Probability*^1^* | Confidence Interval | | Log rank test |
| --- | --- | --- | --- | --- |
|  |  | 95% CI Lower Bound**^1^** | 95% CI Upper Bound**^1^** |  |
| Age at diagnosis |  |  |  |  |
| < 30 days | 98.8 | 97.3 | 100.0 | **<0.001** |
| 31-90 days | 98.6 | 95.9 | 100.0 |  |
| 91-180 days | 98.2 | 94.8 | 100.0 |  |
| > 180 days | 84.6 | 73.9 | 96.7 |  |
| Gender |  |  |  |  |
| Female | 96.5 | 93.5 | 99.6 | 0.44 |
| Male | 97.3 | 94.9 | 99.7 |  |
| Histological subtype |  |  |  |  |
| Cellular | 95.6 | 91.4 | 99.9 | 0.31 |
| Classical | 98.9 | 96.7 | 100.0 |  |
| Mixed | 95.3 | 89.2 | 100.0 |  |
| Period of diagnosis |  |  |  |  |
| 1993_2001 | 95.1 | 91.1 | 99.4 | 0.22 |
| 2002_2010 | 99.3 | 98.0 | 100.0 |  |
| 2011_2019 | 94.6 | 89.6 | 100.0 |  |
| Stage |  |  |  |  |
| Stage I | 98.7 | 96.2 | 100.0 | **0.031** |
| Stage II | 98.1 | 95.9 | 100.0 |  |
| Stage III | 93.2 | 87.0 | 99.9 |  |
| *^1^* 5-Year Overall Survival Rates with 95% Confidence Intervals | | | | |
