# Supplementary material for: Characteristics and outcome of congenital mesoblastic nephroma: A report of 376 patients registered in the SIOP 93-01, SIOP WT 2001, UK-IMPORT, and AIEOP protocols
Source: PLoS One. 2026 May 26;21(5):e0349345. doi: 10.1371/journal.pone.0349345 (PMC13210389; doi:10.1371/journal.pone.0349345)
Supplement: S6 Table — (DOCX) [file pone.0349345.s006.docx]

**Supplementary Table 6. Characteristics and survival of CMN Patients with recurrent disease**

| **Pt** | **Gender** | **Age at diagnosis** *(months)* | **Initial treatment** | **Treatment after surgery** | **Staging** | **Histological subtype** | **Time from diagnosis to relapse** *(months)* | **Site of relapse** | **Relapse therapy** | **Status at last follow up** | **Follow up** *(months)* | **Cause of death** |
| --- | --- | --- | --- | --- | --- | --- | --- | --- | --- | --- | --- | --- |
| 1 | Female | 14 | Preop CT | yes | Stage III | Cellular | 4 | Liver | NA | Death | 7 | Progression |
| 2 | Female | 6 | Preop CT | NA | Stage III | Cellular | 3 | Primary site | NA | Alive | 87 | - |
| 3 | Male | 6 | CN | no | Stage III | Cellular | 3 | NA | NA | Death | 12 | Progression |
| 4 | Male | 3 | CN | no | Stage III | Cellular | 6 | Primary site | NA | Alive | 123 | - |
| 5 | Male | 14 | Preop CT | no | Stage II | Cellular | 9.5 | Lung | AVD/CTX + surgery + RT (15 Gy) | Alive | 83 | - |
| 6 | Female | 2 | CN | NA | Stage III | Cellular | 3 | Primary site | NA | Alive | 91 | - |
| 7 | Female | 24 | Preop CT | no | Stage III | Cellular | 3 | Primary site | VD, ICE + surgery + RT 36 Gy | Death | 14 | Progression |
| 8 | Male | 7 | CN | no | Stage III | Cellular | 5 | Primary site | NA | Death | 26 | Progression |
| 9 | Male | 0 | CN | yes | Stage III | Mixed | 5 | Primary site | Surgery + VA regimen | Alive | 225 | - |
| 10 | Female | 0 | CN | no | Stage II | Mixed | 7 | Primary site | VA regimen | Alive | 90 | - |
| 11 | Male | 4 | CN | no | Stage II | Mixed | 1 | Primary site | NA | Death | 3 | Progression |
| 12 | Male | 7 | CN | no | Stage II | Mixed | 2 | Primary site | NA | Alive | 22 | - |
| 13 | Male | 0 | CN | no | Stage II | Mixed | 6 | Primary site |  | Death | 15 | SIDS |
| 14 | Male | 10 | CN | no | Stage III | Classical | 8 | Testis | Surgery only | Alive | 135 | - |
| 15 | Male | 5 | CN | no | Stage III | Classical | 4 | Primary site | NA | Alive | 23 | - |
| 16 | Female | 32 | Preop CT | no | Stage I | Classical | 9 | Primary site | NA | Death | 18 | Progression |
| 17 | Female | 14 | NA | no | Stage I | Unknown | 3 | Lung | NA | Alive | 204 | - |
| 18 | Male | 1 | CN | NA | Stage III | Unknown | 3 | NA | NA | Alive | 35 | - |

Legend. Preop CT: preoperative chemotherapy; CN: complete nephrectomy ; NA: not available; VA: Vincristine, Actinomycin D; AVD: Actinomycin D, Vincristine, Doxorubicine; ICE: Ifosfamide, Cyclophosphamide, Etoposide; RT: radiotherapy SIDS: Sudden Infant Death Syndrome.
